# Machine learning-based characterization of cuprotosis-related biomarkers and immune infiltration in Parkinson’s disease

**DOI:** 10.3389/fgene.2022.1010361

**Published:** 2022-10-17

**Authors:** Songyun Zhao, Li Zhang, Wei Ji, Yachen Shi, Guichuan Lai, Hao Chi, Weiyi Huang, Chao Cheng

**Affiliations:** ^1^ Department of Neurosurgery, Wuxi People’s Hospital Affiliated to Nanjing Medical University, Wuxi, Jiangsu, China; ^2^ Department of Neurology, Wuxi People’s Hospital Affiliated to Nanjing Medical University, Wuxi, Jiangsu, China; ^3^ Department of Epidemiology and Health Statistics, School of Public Health, Chongqing Medical University, Chongqing, China; ^4^ Clinical Medicine College, Southwest Medical University, Luzhou, China

**Keywords:** PD, cuprotosis, immune cell infiltration, consensus clustering, bioinformatics analysis

## Abstract

**Background:** Parkinson’s disease (PD) is a neurodegenerative disease commonly seen in the elderly. On the other hand, cuprotosis is a new copper-dependent type of cell death that can be observed in various diseases.

**Methods:** This study aimed to identify potential novel biomarkers of Parkinson’s disease by biomarker analysis and to explore immune cell infiltration during the onset of cuprotosis. Gene expression profiles were retrieved from the GEO database for the GSE8397, GSE7621, GSE20163, and GSE20186 datasets. Three machine learning algorithms: the least absolute shrinkage and selection operator (LASSO), random forest, and support vector machine-recursive feature elimination (SVM-RFE) were used to screen for signature genes for Parkinson’s disease onset and cuprotosis-related genes (CRG). Immune cell infiltration was estimated by ssGSEA, and cuprotosis-related genes associated with immune cells and immune function were examined using spearman correlation analysis. Nomogram was created to validate the accuracy of these cuprotosis-related genes in predicting PD disease progression. Classification of Parkinson’s specimens using consensus clustering methods.

**Result:** Three PD datasets from the Gene Expression Omnibus (GEO) database were combined after eliminating batch effects. By ssGSEA, we identified three cuprotosis-related genes ATP7A, SLC31A1, and DBT associated with immune cells or immune function in PD and more accurate for the diagnosis of Parkinson’s disease course. Patients could benefit clinically from a characteristic line graph based on these genes. Consistent clustering analysis identified two subtypes, with the C2 subtype exhibiting higher immune cell infiltration and immune function.

**Conclusion:** In conclusion, our study reveals that several newly identified cuprotosis-related genes intervene in the progression of Parkinson’s disease through immune cell infiltration.

## Introduction

Parkinson’s disease (PD) is the second most common neurodegenerative disease after Alzheimer’s disease, affecting 1.2% of individuals over the age of 65 (Hickman et al., 2018). It is more common in older adults, with an average age of onset of about 60 years, and aging is the greatest risk factor for the development of Parkinson’s disease (Collier et al., 2011). Parkinson’s disease (PD) is a debilitating motor coordination disorder caused by the degeneration of dopamine neurons in the substantia nigra (SN) (Ballance et al., 2019). The main clinical manifestations are resting tremors, bradykinesia, myotonia, and postural gait disturbances (Hammond et al., 2019). Other motor dysfunctions include gait and postural changes, speech and swallowing difficulties, and changes in expression (Zhang et al., 2021). In recent years it has been increasingly noted that non-motor symptoms such as depression, constipation, and sleep disturbances are also common complaints in patients with Parkinson’s disease, and they can have an even greater impact on a patient’s quality of life than motor symptoms (Cederroth et al., 2019). More research is needed on how to prevent motor complications. The exact cause of Parkinson’s disease remains unclear, and genetic factors, environmental factors, aging, and oxidative stress may all be involved in the degenerative death process of PD dopaminergic neurons (Fung et al., 2017). Therefore, early identification of molecular biomarkers of PD is crucial to initiate timely treatment before the onset of motor symptoms.

Copper is an essential trace element that plays an important role in maintaining human life activities, and mechanisms involving copper may represent potential therapeutic targets for different pathologies, and significant changes in its levels in the body may be a potential pathogenic factor in Parkinson’s disease (Atrian-Blasco et al., 2017). Reduced binding of copper to ceruloplasmin in PD patients, resulting in elevated free copper levels, has been shown to be associated with oxidative stress and neurodegeneration (Ajsuvakova et al., 2020). A recent study identified a new mode of cell death that is dependent on and regulated by copper ions in the cell body: cuprotosis. By directly binding to the lipid acylated components of the tricarboxylic acid cycle pathway, copper ions lead to abnormal aggregation of lipid acylated proteins and loss of iron-sulfur cluster proteins, resulting in proteotoxic stress and ultimately cell death (Tsvetkov et al., 2022). Dysregulation of copper homeostasis may trigger cytotoxicity, and changes in intracellular copper levels can ultimately affect the development and progression of neurological diseases (Genoud et al., 2020; Li et al., 2020). In contrast, inhibition of copper transporter protein attenuated α-synuclein-mediated pathological changes in Parkinson’s patients and reduced the increase in proteogenic fibrillation and oxidative stress (Davies et al., 2014; Gou et al., 2021). Also, abnormal tricarboxylic acid cycle function is closely associated with the development of Parkinson’s disease, especially dopamine neurons are much more dependent on mitochondrial metabolism than other cell types (Supandi and van Beek, 2018; Cai et al., 2019). This suggests that inhibiting the occurrence of cuprotosis in neurons through drugs may be a strategy to combat Parkinson’s disease.

In addition, there is growing evidence that the immune system is allied to neuronal death and PD pathogenesis. Recent studies have demonstrated that early stages of Parkinson’s disease progression can be confirmed by detecting immune cell components in the blood, leading to earlier detection and confirmation of the disease (Farmen et al., 2021). Microglia are the brain’s resident immune cells, and activated microglia correlate directly with the clinical and pathological severity of Parkinson’s disease (Lanskey et al., 2018). Current research also includes the function of various immune cells, such as NK cells (Earls and Lee, 2020) and T cells (Yeapuri et al., 2022), but there is still a gap in how these cells play a role in the progression of cuprotosis in PD.

Currently, microarray technology and integrated bioinformatics analysis have been widely used to identify potential novel biomarkers and their roles in various diseases to further explore the pathogenesis and develop potential therapeutic approaches (Zhao et al., 2021). In contrast, there have not been any studies on cuprotosis-related forms of Parkinson’s disease. In this study, four datasets (GSE8397, GSE7621, GSE20163, and GSE20186) were combined into one integrated dataset by the SVA method to eliminate batch differences. To explore the immune cell or immune function correlation of CRGs with PD, ssGSEA was used to study immune infiltration in PD, and consistency clustering analysis was performed to identify pathway differences in cuprotosis-related gene groupings. We believe our findings will provide greater insight into the characterization of cuprotosis progression in PD and provide potential prognostic biomarkers to design rational therapeutic regimens.

## Materials and methods

### Raw data acquisition

Five PD datasets (GSE8397, GSE7621, GSE20163, GSE20186, and GSE42966) were downloaded from the NCBI Gene Expression Omnibus (GEO; https://www.ncbi.nlm.nih.gov/geo/). The above five datasets are all gene expression arrays, GSE7621 generated using GPL570 (HG-U133_Plus_2) Affymetrix Human Genome U133 Plus 2.0 Array. GSE8397, GSE20164, and GSE20186 generated *via* GPL96 (HG-U133A) Affymetrix Human Genome U133A Array was generated. GSE42966 was generated by GPL4133 Agilent-014850 Whole Human Genome Microarray 4 × 44K G4112F. The dataset of GSE8397 included 24 nigrostriatal (SN) samples from PD patients and 15 nigrostriatal samples from normal subjects; GSE20163 contained nine nigrostriatal samples from PD and eight nigrostriatal samples from control subjects; GSE7621 used nine normal nigrostriatal samples from controls and 16 nigrostriatal samples from 16 Parkinson’s disease patients; GSE20186 contained 14 PD nigrostriatal samples and five control samples. GSE42966 served as the validation group and included four Braak3 nigrostriatal samples from patients and five Braak4 patient samples.

### Selection of characteristic genes

Three machine learning algorithms: LASSO regression analysis, random forest, and SVM-RFE (Sanz et al., 2018) were used to screen for eigengenes. LASSO was implemented as a dimensionality reduction method to perform variable screening and complexity adjustment while fitting a generalized linear model. LASSO analysis was implemented with a penalty parameter utilizing a 10-fold cross-verification *via* the “glmnet” package (Engebretsen and Bohlin, 2019). Recursive feature elimination (RFE) in the random forest algorithm is a supervised machine learning method for ranking cuprotosis-associated genes in Parkinson’s disease. Predictive performance is estimated by tenfold cross-validation and genes with relative importance >0.25 are identified as feature genes. SVM-RFE is a small-sample learning method that essentially bypasses the traditional process of induction to deduction and enables efficient “transductive inference” from training to prediction samples, simplifying the usual classification and regression problems.

### Data processing and identification of differentially expressed genes

The four raw datasets were pre-processed by affy in R, including background calibration, normalization, and log2 transformation (Irizarry et al., 2003). When multiple probes correspond to a common gene, their average values were taken as their expression values. In addition, the R package “sva” was used to eliminate batch effects (Buus et al., 2017). The limma package was applied to the four GEO cohorts as a way to screen for differentially expressed cuprotosis-related genes. *p*-values < 0.05 and |log2 Fold change (FC)|>0.2 were set as cut-off points for DEGs (Ritchie et al., 2015). When performing differential analysis of the two PD subtypes, FDR values <0.05 and |logFC|>1 of DEGs were considered to be significantly different.

### Functional enrichment analysis

Functional enrichment analysis, including both Kyoto Encyclopedia of Genes and Genomes (KEGG) and Gene Ontology (GO) analyses, was performed by the “clusterProfiler” package in R software. The BH method was utilized to adjust the *p*-value. Single-sample gene set enrichment analysis (ssGSEA) was used to calculate the infiltration score of 16 immune cells and 13 immune-related pathways by the “gsva” package in R software (Rooney et al., 2015). Finally, we also examined the correlation between cuprotosis-related genes and immune cells and immune function in Parkinson’s disease samples.

### Gene set enrichment analysis

Gene set enrichment analysis is a computational method used to test whether genes show statistically significant and consistent changes between two biological states. The most significant relevant signaling pathways are identified by 10,000 alignment tests. A corrected *p*-value of less than 0.05 and a false discovery rate (FDR) of less than 0.05 was used as criteria. Finally, we selected the top 5 KEGG pathways for statistical analysis and ridge mapping using the R package “clusterPro”.

### Consensus clustering

Consensus clustering is used to calculate how many unsupervised classes there are in a dataset. The consensus clustering (CC) method was used. Based on the ICI characteristics, we used the R package “ConsensusClusterPlus” (Wilkerson and Hayes, 2010) to classify Parkinson’s patients in GSE8397, GSE7621, GSE20163, and GSE20186 into different ICI clusters. These results are displayed after being run 1,000 times to verify the accuracy and reproducibility of the program, and we use the heat map function of the R language. Consensus matrix plots, consensus cumulative distribution function (CDF) plots, the relative change in area under the CDF curve, and trace plots were used to find the optimal number of clusters.

### Gene set variation analysis

GSVA is a non-parametric unsupervised analysis method that is mainly used to assess the results of gene set enrichment in microarrays and transcriptomes. It is mainly used to assess whether different metabolic pathways are enriched between samples by converting the gene expression matrix between samples into the expression matrix of gene sets between samples (Hoang et al., 2019). Fifty signature gene sets were selected from MSigDB as reference sets. The GSVA package and its ssGSEA function were used to obtain the GSVA score for each gene set. The GSVA score indicates the absolute enrichment of each gene set. The Limma package was used to compare the differences in GSVA scores per genome between subtypes.

### Statistical analysis

All analyses were performed using R version 4.1.1, 64-bit6, and its support package. The nonparametric Wilcoxon rank sum test was used to test the relationship between two groups of continuous variables. Correlation coefficients were examined using spearman correlation analysis. In all statistical investigations, *p* < 0.05 was considered statistically significant. The “rms” package was used to merge the characteristic genes to create a nomogram. Calibration curves were used to assess the accuracy of the nomogram. The clinical utility of the column line graphs was evaluated by decision curve analysis. PCA plots were described using the ggplot2 package.

## Results

### Identification of CRGs

**FIGURE 1:**
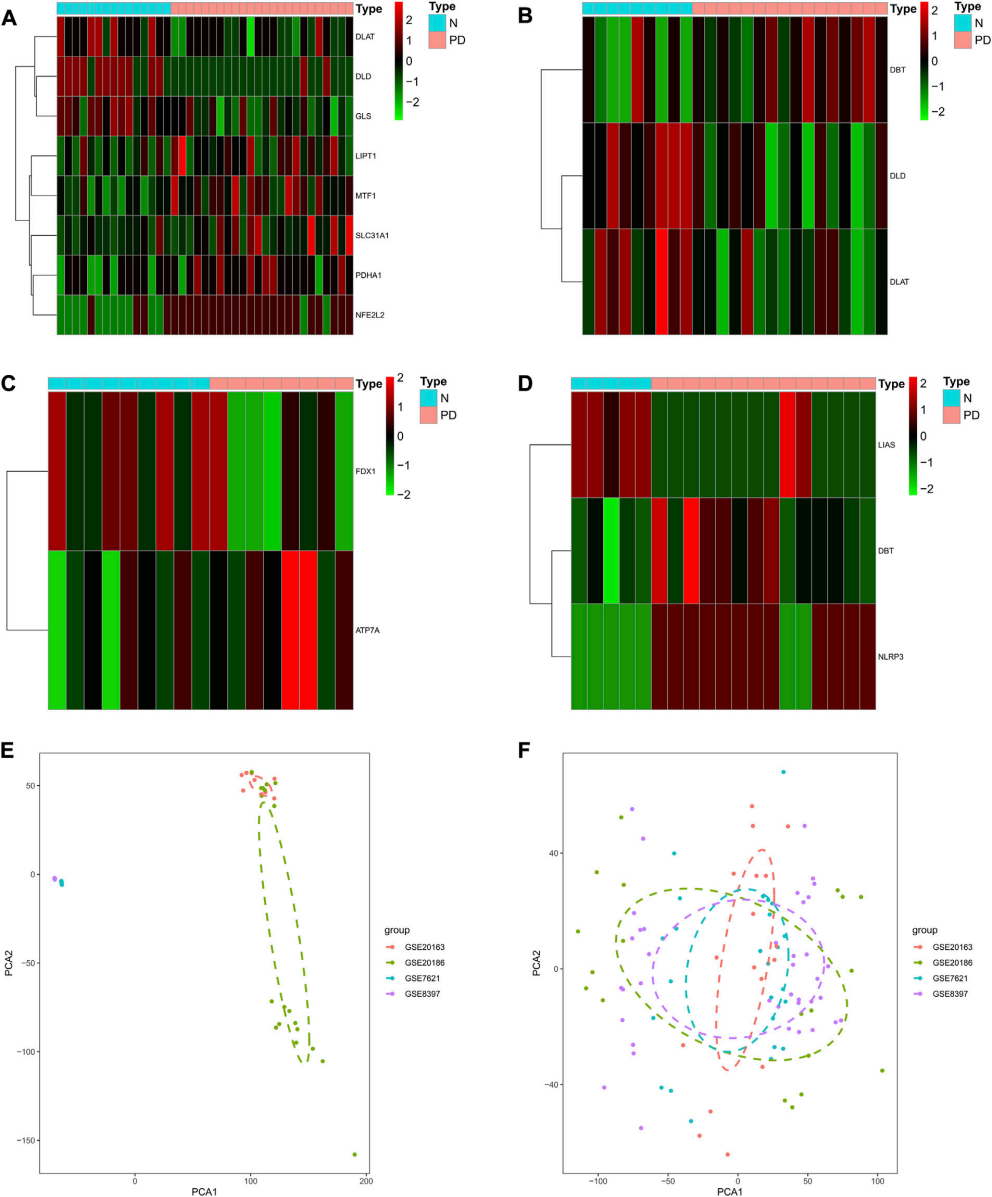
Identification of Parkinson’s onset and cuprotosis-related genes in the combined expression profile of the GEO cohort. **(A–D)** Heat map showing differentially expressed CRGs for the GSE8397, GSE7621, GSE20163, and GSE20186 cohorts. **(E)** PCA plot showing the combinatorial expression profile of the GEO cohort. **(F)** PCA plot showing the combined expression profile of the GEO cohort after batch effect.

First, using the limma package to perform differential analysis of CRGs in the four GEO cohorts PD and control, respectively (Figures 1A–D), we found that DLD, DLAT, and DBT were differentially expressed in GSE7621, NFE2L2, DLD, MTF1, GLS, DLAT, PDHA1, PDHA1, and LIPT1 were differentially expressed in GSE8397. SLC31A1, FDX1, and ATP7A were differentially expressed in GSE20163, while NLRP3, LIAS, and DBT were differentially expressed in GSE20186. To investigate the role of cuprotosis-related genes in the progression of Parkinson’s disease, we combined the expression profiles of 38 normal brain substantia nigra and 62 brain substantia nigra specimens from the GSE8397, GSE7621, GSE20163, and GSE20186 cohorts of Parkinson’s patients (Figure 1E), which were batch processed for subsequent analysis (Figure 1F).

### Assessment of the microenvironment in Parkinson’s disease

**FIGURE 2:**
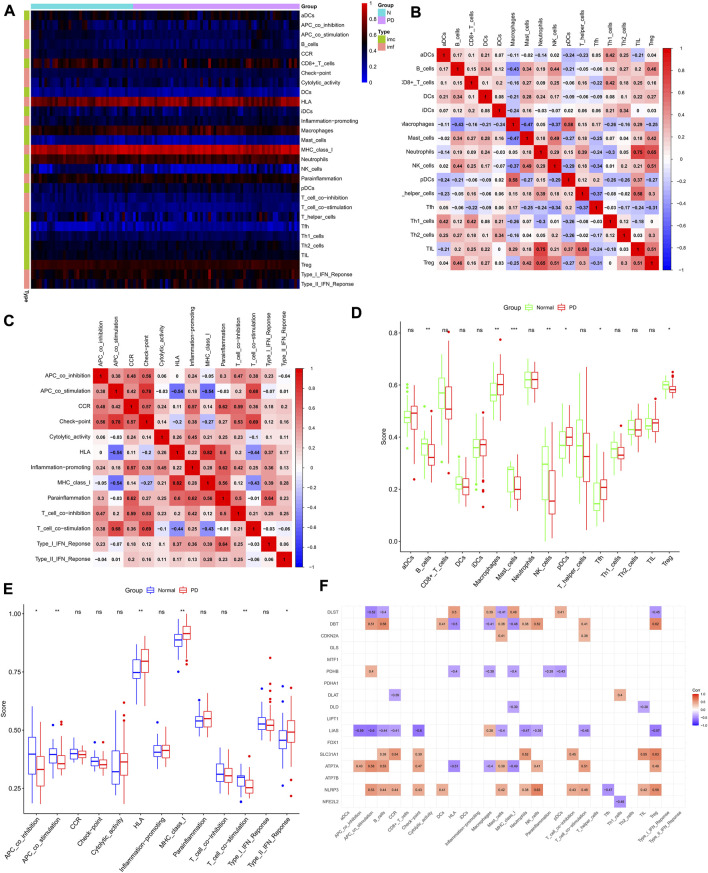
Immune cell infiltration analysis. **(A)** Heat map of immune cells and immune function in PD group and normal control group. **(B,C)** Correlation matrix of immune cells and immune function. The red color indicates a positive correlation, the blue color indicates a negative correlation, and the darker color indicates a stronger correlation. **(D,E)** Comparison of the degree of immune cell infiltration and immune function between the PD group and normal control group. **(F)** Correlation analysis of cuprotosis-related genes and immune cells as well as immune function. *p < 0.05, **p < 0.01, ***p < 0.001, ns no significance.

We quantified the ssGSEA enrichment scores for different immune cell subpopulations, related functions or pathways in PD, and normal controls. The abundance of immune cells and immune functions in each sample is shown in the heat map (Figure 2A). Figures 2B,C show the correlation heat map between immune cells and immune function, with the darker red color representing a larger association between the two. We compared ssGSEA scores between PD and normal groups and showed that B cells, mast cells, NK cells, and regulatory T cells were more abundant in normal brain substantia nigra tissue, while macrophages, pDCs, and Tfh were more abundant in PD substantia nigra (Figure 2D). Human leukocyte antigen, MHC class_I, and type II interferon responses were higher in the PD group (Figure 2E), while APC_co_inhibition, APC_co_stimulation, and T_cell_co-stimulation were enriched in the normal group.

We then collected 17 reported cuprotosis-related genes, and we showed the correlation between these genes and immune pathways in ssGSEA results using a heat map (Figure 2F). We found that the vast majority of CRGs act in the immune microenvironment of PD.

### Selection of characteristic genes *via* least absolute shrinkage and selection operator, random forest, and support vector machine-recursive feature elimination algorithms

**FIGURE 3:**
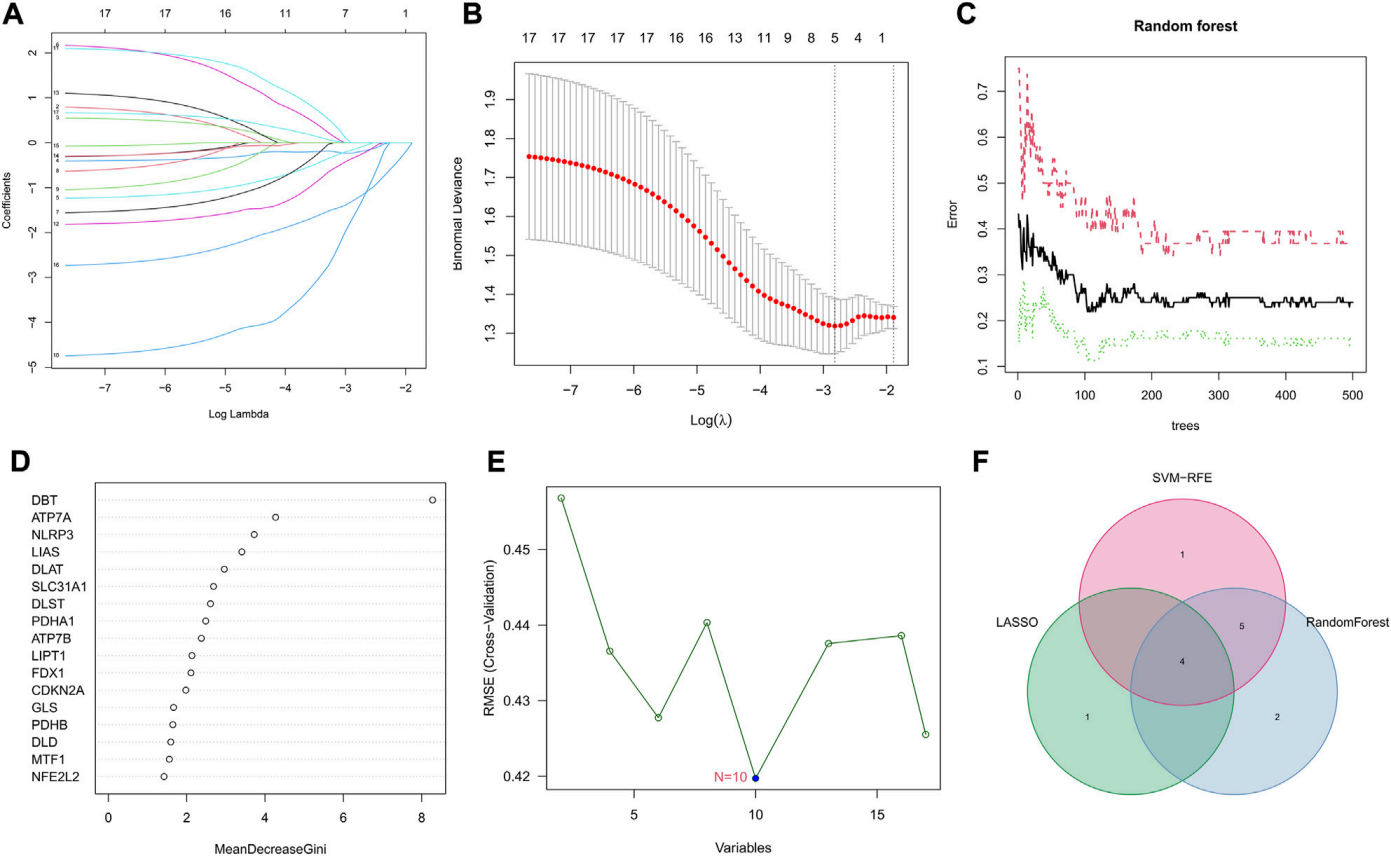
Selection of signature genes among genes associated with Parkinson’s onset and cuprotosis. **(A)** Ten cross-validations of adjusted parameter selection in the LASSO model. Each curve corresponds to one gene. **(B)** LASSO coefficient analysis. Vertical dashed lines are plotted at the best lambda. **(C)** Relationship between the number of random forest trees and error rates. **(D)** Ranking of the relative importance of genes. **(E)** SVM-RFE algorithm for feature gene selection. **(F)** Venn diagram showing the feature genes shared by LASSO, random forest, and SVM-RFE algorithms.

Three machine learning algorithms were applied to select signature genes among genes associated with Parkinson’s disease onset and cuprotosis. Five variables, ATP7A, SLC31A1, DLAT, PDHB, and DBT, were identified as diagnostic markers for PD by the LASSO regression operation (Figures 3A,B). Figure 3C represents the effect of the number of decision trees on the error rate. The *x*-axis represents the number of decision trees, while the *y*-axis represents the error rate. The error rate is usually stable when we use about 104 decision trees. For the random forest algorithm, 11 signature genes with relative importance scores greater than two were identified, including DBT, ATP7A, NLRP3, LIAS, DLAT, SLC31A1, DLST, PDHA1, ATP7B, LIPT1, and FDX1 (Figure 3D). For the SVM-RFE algorithm, the error was minimized when the number of features was 10, including DBT, ATP7A, LIAS, NLRP3, DLST, SLC31A1, DLAT, ATP7B, MTF1, and PDHA1 (Figure 3E). After the intersection, four common signature genes, ATP7A, SLC31A1, DLAT, and DBT, were finally identified (Figure 3F).

### Diagnostic efficacy of characteristic genes

**FIGURE 4:**
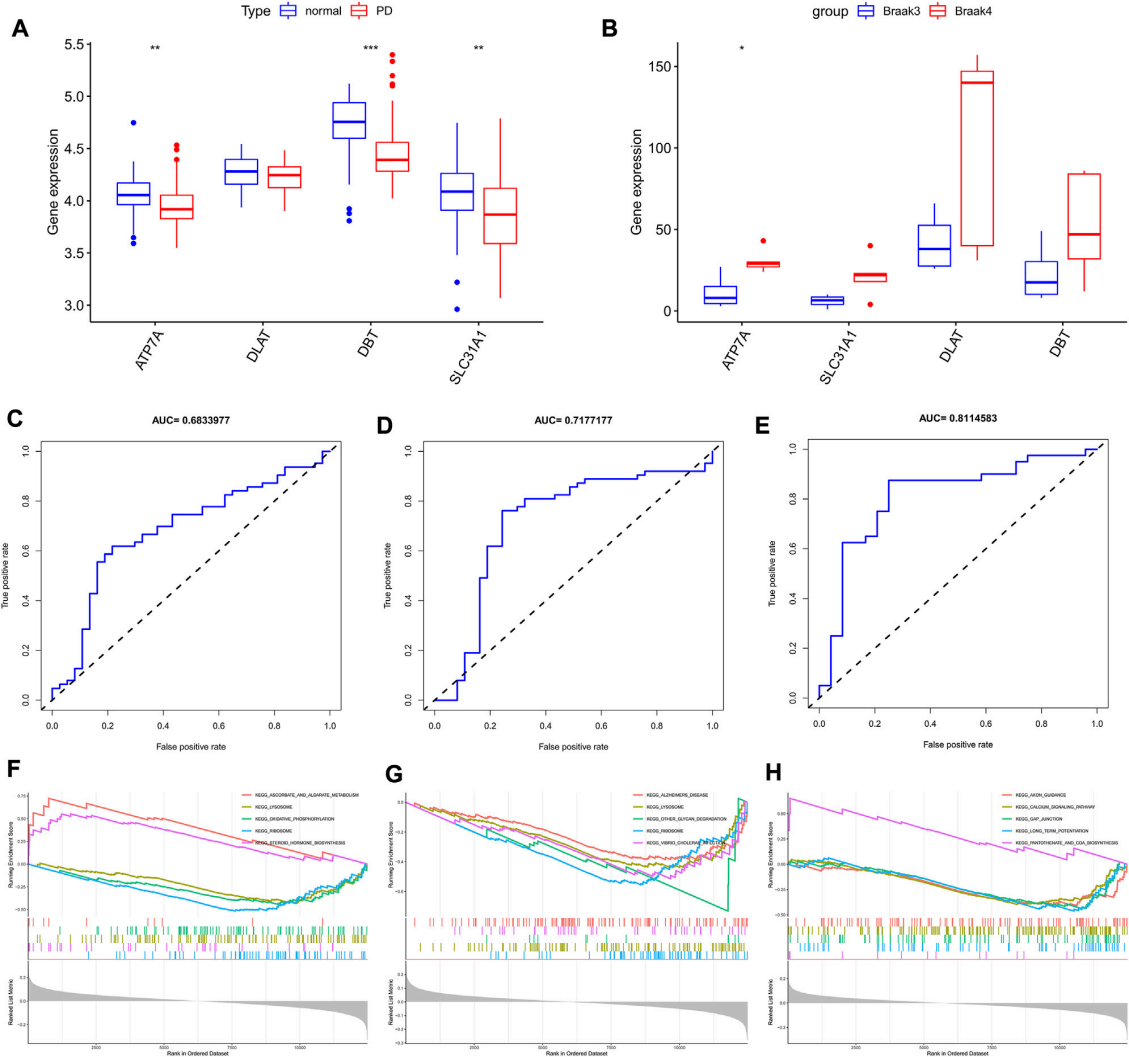
Characterized gene expression, diagnostic efficacy, and enrichment analysis. **(A)** Box line plot depicting trait gene expression in Parkinson’s disease and normal controls. **(B)** Box line plot depicting trait gene expression in braak3 and braak4 phases. **(C–E)** ROC curves for estimating the diagnostic performance of the signature genes. **(F–H)** GSEA identifies the major signaling pathways involved in signature genes. *p < 0.05, **p < 0.01, ***p < 0.001.

In the four combined GEO cohorts, the expression of the three characteristic genes ATP7A, SLC31A1, and DBT was lower in PD than in normal controls (Figure 4A, *p* < 0.05), while DLAT was not significantly different in the two groups. In contrast, in the comparison between stage IV and V Parkinson’s disease patients, probably due to the small sample size, only ATP7A was significantly different in the two groups (Figure 4B, *p* < 0.05), suggesting what seems to indicate their potential role in Parkinson’s onset and progression. Based on the results of the analysis of variance, we estimated the diagnostic performance of the three signature genes. The AUC values of the ROC curves for the signature genes were 0.683 for ATP7A (Figure 4C), 0.717 for DBT (Figure 4D), and 0.811 for SLC31A1 (Figure 4E), respectively. With GSEA, we evaluated the signaling pathways involved in the signature genes. Our results show that ATP7A (Figure 4F) is associated with steroid hormones, DBT is mainly associated with Alzheimer’s disease (Figure 4G), and SLC31A1 (Figure 4H) is associated with axon guidance, calcium signaling pathways, and Long-term potentiation.

### Establishment of nomogram for predicting Parkinson’s disease

**FIGURE 5:**
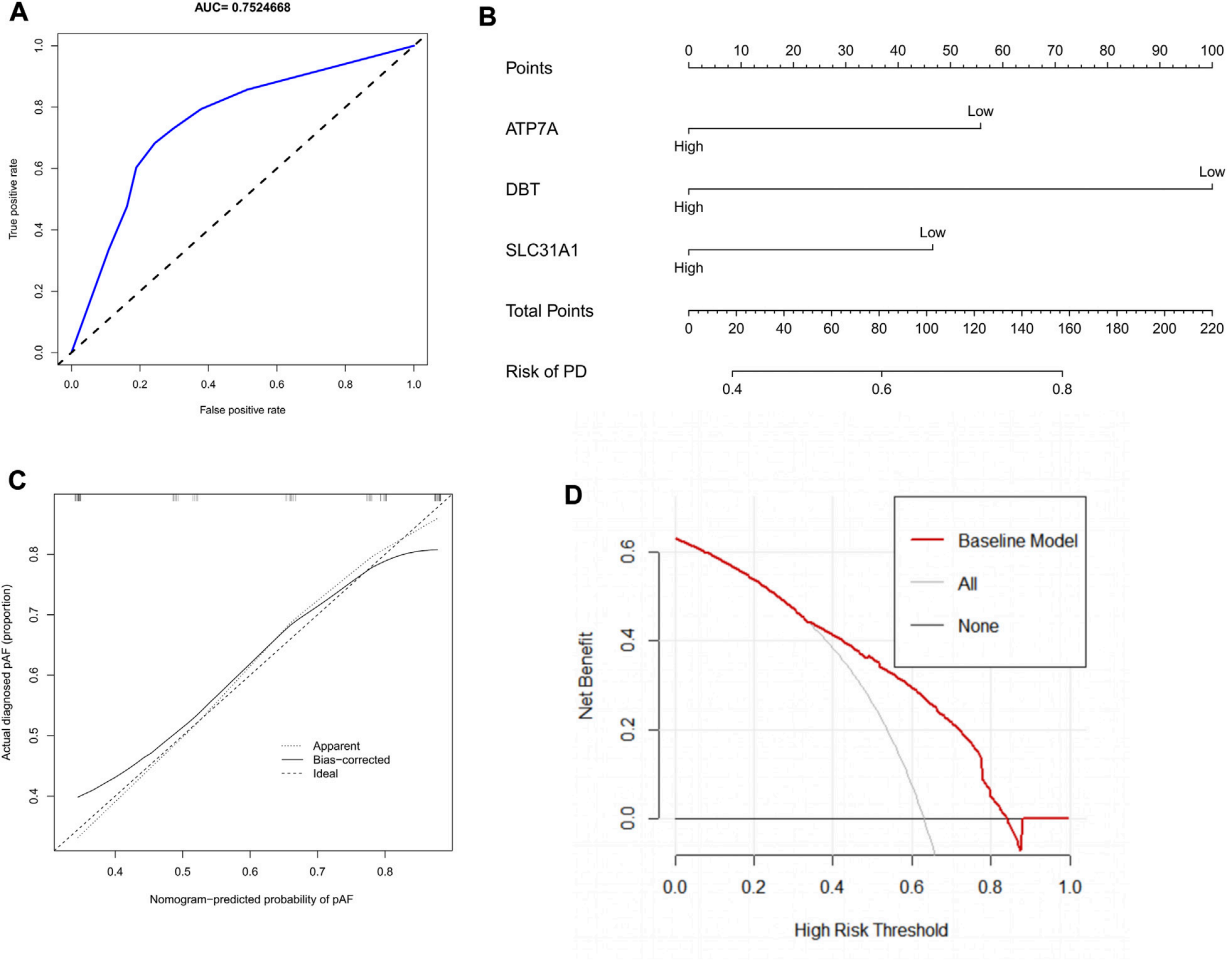
Construction of column line graph based on Characteristic CRGs. **(A)**The ROC curves estimating the diagnostic performance of characteristic genes. **(B)** Construction of column line graph integrating Characteristic CRGs for PD. in the column line graph, each variable corresponds to a score, and the total score can be calculated by summing the scores of all variables. **(C)** Calibration curves to estimate the prediction accuracy of the column line graphs. **(D)** Decision curve analysis showing the clinical benefit of column line graphs.

When these three variables were integrated into one variable, the AUC of the ROC curve was 0.752 (Figure 5A). This suggests that the three characteristic CRGs have good diagnostic efficiency in predicting Parkinson’s disease progression. Columnar line graphs were constructed to diagnose Parkinson’s disease by integrating trait genes and clinical traits (Figure 5B). In the column line graph, each trait gene corresponds to a score, and the total score is obtained by summing the scores of all trait genes. The total score corresponds to the different risks of Parkinson’s. The calibration curves showed that the column line plot was able to accurately estimate the prediction of Parkinson’s onset (Figure 5C). As shown in the decision curve analysis, patients with Parkinson’s can benefit from the column line graph (Figure 5D).

### Identification of immune-associated cuprotosis genes subtypes in parkinson’s disease

**FIGURE 6:**
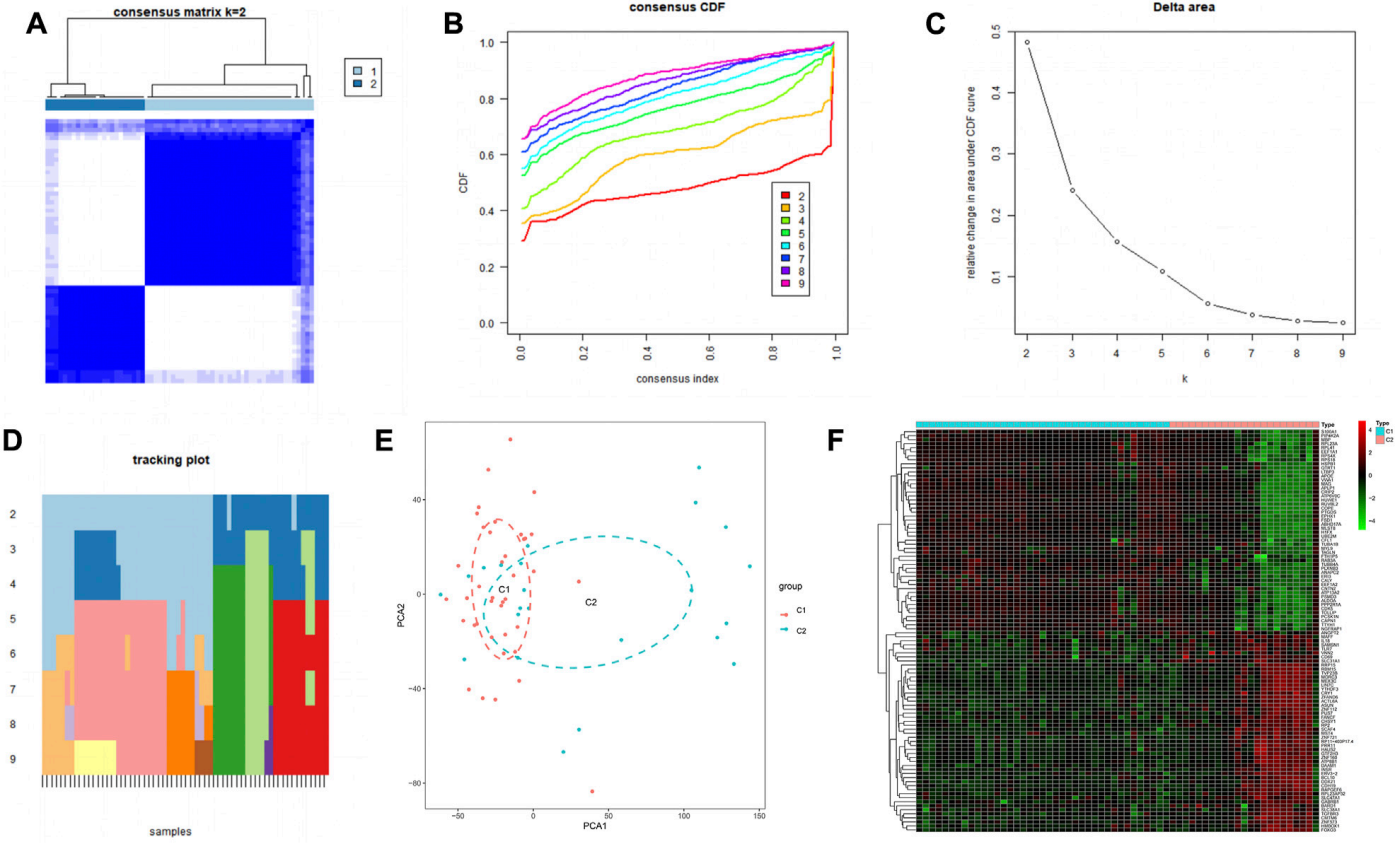
Construction of two subpopulations based on cuprotosis-related genes in the GEO cohort. **(A)** Heat map of the consensus matrix at k = 2. **(B)** Consensus CDF at k = 2–9. **(C)** Relative change in area under the CDF curve. **(D)** Trace plot of sample classification when k = 2–9. **(E)** 3DPCA plot showing that cuprotosis-associated genes effectively classify Parkinson’s patients into two subgroups (C1 and C2). **(F)** Heat map showing differential gene expression in the two immune subtypes.

PD samples were clustered by the consensus clustering method based on the expression profiles of three cuprotosis signature genes. The optimal number of subtypes was 2 as determined by consensus matrix plots, CDF plots, relative changes in regions under the CDF curve, and trace plots (Figures 6A–D). The two immune subtypes were named C1 and C2. PCA demonstrated significant differences between the subtypes (Figure 6E). The heat map (Figure 6F) shows the differential gene expression in the two immune subtypes.

### Different immunological characteristics of the two subtypes

**FIGURE 7:**
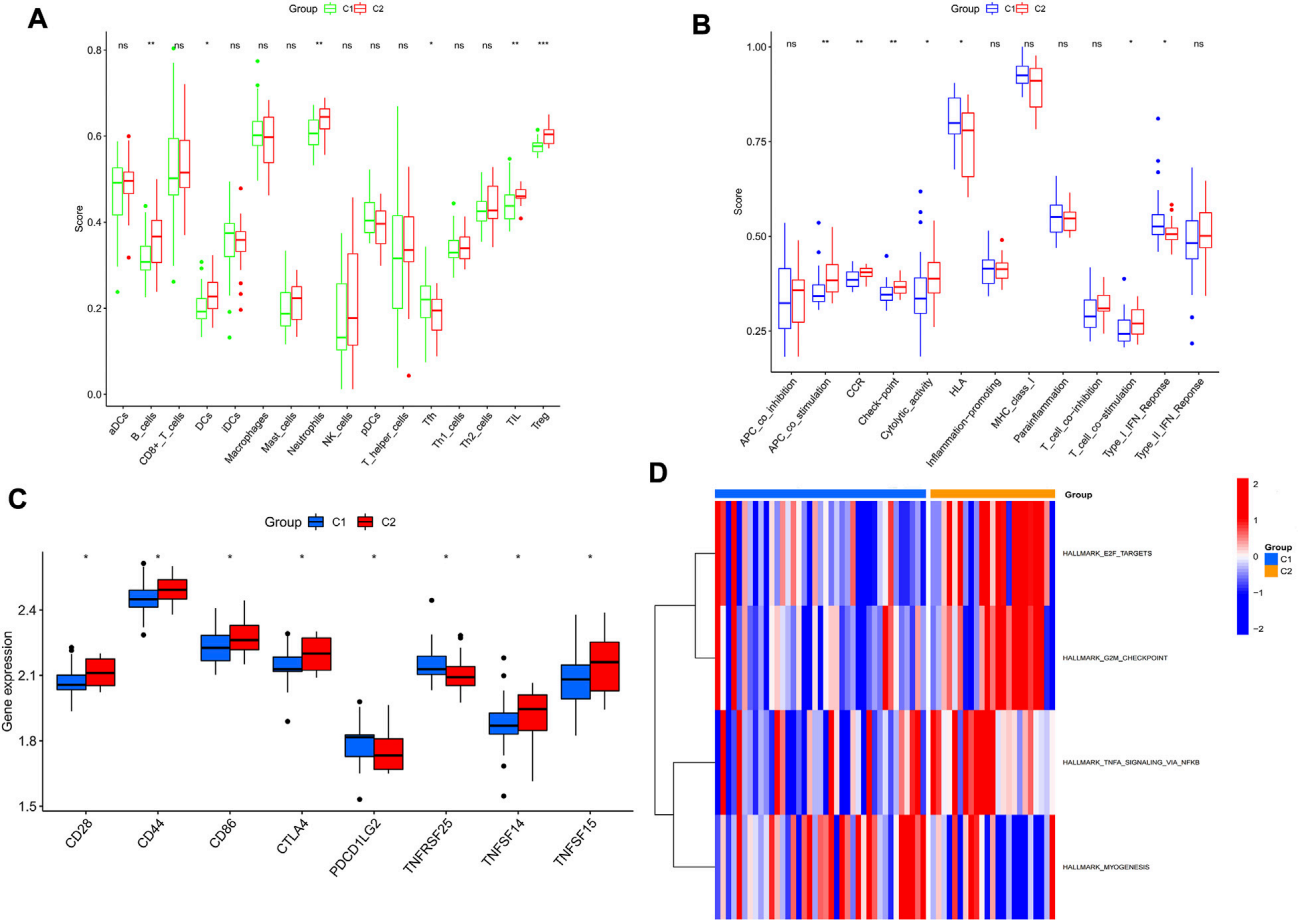
The two subtypes have different immunological features and molecular mechanisms. **(A,B)** Comparison of the degree of immune cell infiltration and immune function between the two subtypes. **(C)** Box plot showing the mRNA expression of signature genes in the two subtypes. **(D)** Heat map showing the level of enrichment of the set of signature genes in the two subtypes. *p < 0.05; **p < 0.01 and ***p < 0.001.

As shown in Figures 7A,B, the C2 subtype had higher immune functions such as B_cells, DCS, Neutrophils, TIL and Treg, APC_co_stimulation, CCR, and Check-point than the C1 subtype. Most of the immune checkpoint genes such as CTLA4 and CD28 were also expressed more in the C2 subtype than in the C1 subtype (Figure 7C). GSVA results showed that TNFA_SIGNALING signals, G2/M cell cycle checkpoints, and E2F transcriptional genes (Figure 7D) were higher in the C2 subtype than in the C1 subtype. Overall, C2 could be identified as an immune subtype and C1 as a non-immune subtype.

## Discussion

Parkinson’s disease is a severe neurodegenerative disorder. The typical pathology of Parkinson’s disease is characterized by the loss of dopaminergic neurons in the dense substantia nigra and the aggregation of alpha-synuclein, forming Lewy vesicles and Lewy synapses. However, the exact pathogenesis of PD is currently unknown. To our knowledge, no previous studies have examined the correlation between CRG and the development of Parkinson’s disease. Surprisingly, many CRGs are differentially expressed between the nigrostriatal and normal brain tissue in Parkinson’s disease, and most of these genes are significantly associated with immune function and likely influence the staging of Parkinson’s disease, suggesting a potential role of cuprotosis in Parkinson’s disease.

Investigations have found a higher incidence of Parkinson’s disease in areas with higher copper emissions. But the role of copper in Parkinson’s disease is controversial, as some evidence suggests the need to increase copper levels, while other results suggest the opposite (Baldari et al., 2020). The main role of copper is mediated by its ability to trigger, maintain and even enhance free radical production. In general copper binding to α-synuclein triggers increased proteogenic fibrillation and oxidative stress (Gou et al., 2021). However, under the influence of copper cyanobactin (Prohaska, 2011), the reduction of copper may be associated with iron accumulation, while iron deposition and consequent ferroptosis may be an important mechanism of dopaminergic neuronal death in PD (Wang et al., 2022). In an interesting *in vitro* study (Spencer et al., 2011), complexes formed by dopamine oxidation products with copper caused severe damage to DNA. By injecting copper sulfate directly into the substantia nigra of mice, a decrease in dopamine, an increase in oxidative stress, and a loss of immune response were directly induced (Yu et al., 2008). This also suggests that the inhibition of cuprotosis combined with immunotherapy will be the focus of treatment for Parkinson’s patients.

An investigation pointed out that the enrichment of senescent cells in tissues is associated with disorders of tissue homeostasis, including Alzheimer’s and Parkinson’s, and that copper accumulation is a common feature of senescent cells *in vitro* (Masaldan et al., 2018). In addition to this, ferroptosis inhibitors (iron chelators) have demonstrated good clinical relief of PD symptoms, whereas the clinical translation of copper chelators in PD has not progressed (Nunez and Chana-Cuevas, 2018). Treatment strategies for Parkinson’s disease must be adopted with caution due to the delicate balance of copper homeostasis.

Among the 38 PD and 62 normal samples in the GSE8397, GSE7621, GSE20163, and GSE20186 datasets, we selected three signature genes (ATP7A, SLC31A1, and DBT) based on three machine learning algorithms. These three genes were differentially expressed in the PD and control groups and most likely influenced the Braak staging of PD. All this evidence can indicate the role of the signature genes in Parkinson’s disease. The signature genes involved in this study include ATP7A, SLC31A1, and DBT. ATP7A is widely recognized as a copper-transporting ATPase due to mutations in its gene that cause impaired copper transport and further cause the neurological genetic disorder Menkes disease (Li et al., 2018). ATP7A is involved in axonal growth, synaptic integrity, and neuronal activation and has an important role in the root of stability for neurological function (Kaler, 2011). The SLC31A1 (solute carrier family 31 member 1) gene, also known as CTR1 (copper transporter protein 1), encodes a high-affinity copper transporter protein in cell membranes that act as a homotrimer to influence dietary copper uptake. Its more studied in tumors, such as pancreatic cancer (Yu et al., 2019), colorectal cancer (Barresi et al., 2016), and lung cancer (Barresi et al., 2016), as a means of copper depletion affecting the prognosis of cancer patients. DBT is a component of the branched-chain α-keto acid dehydrogenase complex, and its deficiency allows the accumulation of branched-chain amino acids and their harmful derivatives in the body (Podebrad et al., 1999). An association between Alzheimer’s disease and Parkinson’s disease and the 2-oxoglutamate dehydrogenase gene has been reported (Hengeveld et al., 2002).

We constructed two isoforms from three cuprotosis genes based on machine learning and immune expression profiles. The C2 subtype exhibited higher immune cell infiltration and immune function compared to the C1 subtype. Therefore, our classification reflects the immune status of Parkinson’s disease, which may help in the diagnosis and treatment of PD. Although machine learning algorithms can identify cuprotosis-related genes in the characterization of Parkinson’s immune progression, experiments are still needed to further elucidate the mechanisms of the characterized genes.

## Conclusion

Our results identified three characteristic cuprotosis-related genes ATP7A, SLC31A1, and DBT involved in the immune process of Parkinson’s disease. In addition, Parkinson’s disease samples were classified into immune and non-immune subtypes by a new molecular classification. However, little is known about the relationship between specific genes and PD, and must be performed *in vitro* and *in vivo* to verify our conjectures. This study provides important information to elucidate the physiological and pathological processes of cuprotosis in PD. Overall, our findings may contribute to the design of better immunotherapies for Parkinson’s disease based on the mechanisms of cuprotosis.

## Data Availability

Publicly available datasets were analyzed in this study. This data can be found here: The datasets analyzed in the current study are available in the GEO (https://www.ncbi.nlm.nih.gov/geo/). All raw data and original images can be found in the jianguoyun (https://www.jianguoyun.com/p/DQEJLh0Q0pH7ChjCutoEIAA).
